# Identification and expression analysis of the Xyloglucan transglycosylase/hydrolase (*XTH*) gene family under abiotic stress in oilseed (*Brassica napus* L.)

**DOI:** 10.1186/s12870-024-05121-5

**Published:** 2024-05-15

**Authors:** Jingdong Chen, Heping Wan, Huixia Zhao, Xigang Dai, Wanjin Wu, Jin Liu, Jinsong Xu, Rui Yang, Benbo Xu, Changli Zeng, Xuekun Zhang

**Affiliations:** 1 MARA Key Laboratory of Sustainable Crop Production in the Middle Reaches of the Yangtze River (Co-construction by Ministry and Province), College of Agriculture, Yangtze University, Jingzhou, 434025 China; 2 Hubei Engineering Research Center for Protection and Utilization of Special Biological Resources in the Hanjiang River Basin, College of Life Science, Jianghan University, Wuhan, 430056 Hubei China

**Keywords:** *Brassica napus*, XTH gene family, Abiotic stress tolerance, Expression profiling

## Abstract

*XTH* genes are key genes that regulate the hydrolysis and recombination of XG components and plays role in the structure and composition of plant cell walls. Therefore, clarifying the changes that occur in *XTHs* during plant defense against abiotic stresses is informative for the study of the plant stress regulatory mechanism mediated by plant cell wall signals. XTH proteins in *Arabidopsis thaliana* was selected as the seed sequences in combination with its protein structural domains, 80 members of the *BnXTH* gene family were jointly identified from the whole genome of the *Brassica napus* ZS11, and analyzed for their encoded protein physicochemical properties, phylogenetic relationships, covariance relationships, and interoperating miRNAs. Based on the transcriptome data, the expression patterns of *BnXTHs* were analyzed in response to different abiotic stress treatments. The relative expression levels of some *BnXTH* genes under Al, alkali, salt, and drought treatments after 0, 6, 12 and 24 h were analyzed by using qRT-PCR to explore their roles in abiotic stress tolerance in *B. napus*. *BnXTHs* showed different expression patterns in response to different abiotic stress signals, indicating that the response mechanisms of oilseed rape against different abiotic stresses are also different. This paper provides a theoretical basis for clarifying the function and molecular genetic mechanism of the *BnXTH* gene family in abiotic stress tolerance in rapeseed.

**Supplementary Information:**

The online version contains supplementary material available at 10.1186/s12870-024-05121-5.

## Introduction

In complex and changeable natural environments, crops may be exposed to abiotic stresses such as temperature, drought, salt, heavy metals, etc., potentially compromising their yield and quality [1]. As the first barrier, the cell wall plays an important role in the defense of plants against external environmental stresses [2].

The cell wall has a complex structure consisting of pectin embedded microfibrils and non-cellulosic neutral polysaccharides, crosslinked with structural proteins and, depending on the tissue and organ, with lignin [3]. Plant cell walls are divided into the intercellular layer, primary and secondary walls [4]. The main component of the intercellular layer is pectin, which is located between two neighboring cells and is able to adhere to them and buffer intercellular extrusion [5]; the primary wall is mainly composed of cellulose, hemicellulose, and structural proteins, which has a large plasticity, which allows the cell to maintain a certain shape, but also can be extended with the growth of the cell [6]; the secondary wall, which is mainly composed of cellulose and often contains lignin, located between the plasma membrane and the primary wall, is usually thicker and harder, giving the cell wall great mechanical strength [7].

Xyloglucan (XG) is the main chain component of the polysaccharide hemicellulose that accounts for the largest proportion in the primary cell wall of dicotyledonous plants [8]. The glucan backbone of XG is composed of β-1,4-D-Glup, and the O-6 position is connected to α-D-Xylp. These Xyls can be further modified by other glycosyl groups [9]. The structures of XG are diverse, showing variability among plants, organs, tissues and even within the same plant [10]. The basic function of XG is to bind to the outside of cellulose fibrils to form a load-bearing network of the primary cell wall, which determines the tension of the cell wall. The modification and reconstruction of XG can adjust the elasticity and strength of the cell wall skeleton [11].

The recombination of XG requires the participation of Xyloglucan transglycosylase/hydrolase (XTH) of the GH16 family. XTH has dual activities of Xyloglucan endotransglycosylase (XET) and Xyloglucan hydrolase (XEH), allowing it to independently complete the cleavage and recombination process of XG, and can carry out the catalytic reaction of extending the XG chain without the direct involvement of ribose [12]. *XTH* genes play roles in plant growth and development, especially in response to abiotic stress. The cell wall modification of *xth19* could affect freezing tolerance after cold and sub-zero acclimation. Compared with the Col-0 wild type, the cell wall structure and composition of the *xth19* mutant of Arabidopsis changed, which resulted in a lower freezing tolerance after cold and sub-zero acclimation [13]. Compared to the control, the expressions of *TaXTHs* of wheat were significantly altered under drought stress; the drought tolerance of transgenic plants of Arabidopsis overexpressing *TaXTH12.5a* was improved, with the germination rate, root length, hypocotyl length, and the number of green leaves during the germination stage and nutrient growth stage significantly higher than that of the control lines [14]. Under salt stress, there were 11 differentially expressed *PtrXTHs* in the roots of poplar, nine differentially expressed *PtrXTHs* in the stems, and 7 differentially expressed *PtrXTHs* in the leaves [15]; the water retention capacity of the leaves in transgenic plants of tobacco overexpressing *PeXTH* of Populus were improved, and the net photosynthetic rate had a significant enhancement, which enhanced the salt tolerance of tobacco, indicating that the *XTHs* might play roles for plants in the response to salt stress [16]. It was found that many *XTHs* of soybean responded to ethylene and flooding treatments, The Arabidopsis thaliana AtXTH31 gene is overexpressed in soybean, and transgenic soybean plants with *AtXTH31* overexpressing under flooding stress tolerance showed higher germination rate, and longer roots/ hypocotyls compared to the control lines at the seedling and nutrient growth stages, it was confirmed that *XTHs* played roles in regulating the response of soybean to abiotic stresses [17]. Du et al. found that aluminum (Al) induces *ZmXTHs* to be up-regulated in the maize root system, especially in the root tips, and this induction was Al^3+^ specific; *ZmXTHs* overexpressing plants of Arabidopsis grew more healthily with lower Al content in their roots and root cell walls under Al stress compared to wild-type, suggesting that *ZmXTHs* could be endowed with aluminum tolerance on transgenic Arabidopsis plants by reducing the accumulation of aluminum in roots and cell walls [18]. *XTHs* also played important roles in the response of heavy metal stress in plants: most *XTHs* in ramie (*Boehmeria nivea*) were responsive to cadmium stress, while heterologous expression of BnXTH3, BnXTH6 and BnXTH15 significantly enhances the cadmium tolerance of transgenic yeast cells [19]; Xuan et al. identified 44 possible *MtXTHs* in the *Medicago truncatula* genome, 28 of which were responsive to HgCl_2_, and copper/mercury stresses would significantly induce the expression of the *MtXTH3* both in the roots and shoots. In addition, the expression of *MtXTH3* showed an increasing trend with the elevation of Cu and Hg concentrations [20].

While the impact of XTHs on plant cell wall composition and abiotic stress resilience has been thoroughly investigated in Arabidopsis and various crops, the expression profiles of these genes under abiotic stress conditions in *Brassica napus* remain comparatively underexplored. In this study, we undertook a comprehensive genome-wide identification of the *BnXTH* gene family members in rapeseed; additionally, we conducted an exhaustive analysis encompassing protein characteristics, gene structure, phylogenetic relationships, and collinearity the expression patterns of *BnXTHs* in two varieties of rapeseed were compared under alkaline (Na_2_CO_3_) stress using RNA-seq analysis. At the same time, by qPCR, the relative expressions of *BnXTHs* under aluminum (AlCl_2_), alkali (Na_2_CO_3_), salt (NaCl) and drought (PEG 6000) stress at four time points (0, 6 h, 12 h and 24 h) were examined, which provided evidence for the response of *BnXTHs* to abiotic stress. The findings of our study establish a foundational framework for subsequent investigations into the functional roles of these genes in the abiotic stress response of rapeseed. Furthermore, they offer a theoretical underpinning for the selection and breeding of abiotic stress-tolerant rapeseed germplasms.

## Results

### Identification and chromosomal localization of the BnXTH gene family

**Fig. 1:**
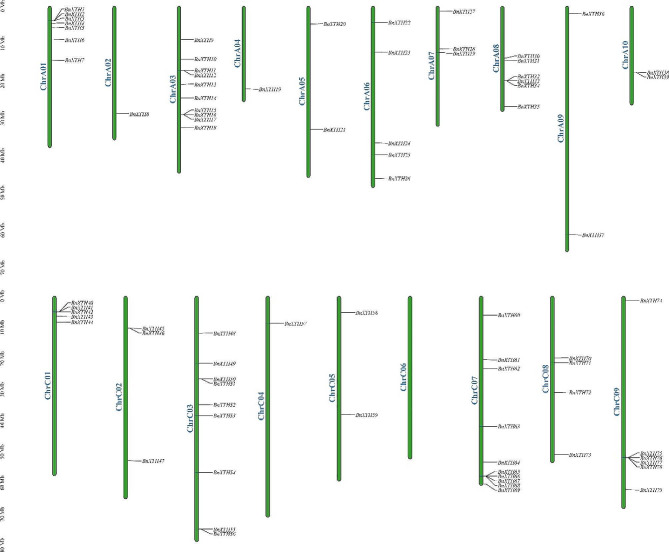
Chromosomal location of the *BnXTHs* in the *B. napus* genome

Joint identification was conducted using BLASTP and HMMER, and the NCBI-CDD tool was used to remove the sequences with incomplete structural domains. Finally, 80 BnXTH proteins were obtained, which were named *BnXTH1* – *BnXTH80* based on the order of the location of their encoding genes on the chromosomes. Among these 80 genes, 79 were mapped to 18 identified chromosomes (Fig. 1), and one was mapped to pseudochromosome Scaffold0027. Of these, 10 *BnXTHs* were localized on ChrA03 and ChrC07, nine on ChrC09, seven on ChrA01, six on ChrA08 and ChrC09, five on ChrA06 and ChrC01, three on ChrA07, ChrC02, ChrA05, and ChrA09, two were localized on ChrA10 and ChrC05, one was localized on ChrA02, ChrA04, and ChrC04, while no possible *BnXTHs* were detected on ChrC06. Number of *BnXTHs’* exons ranged from one to eight, and the information of their positions in the chromosomes was provided on Table S1.

### Physicochemical properties and subcellular localization prediction of BnXTH proteins

By predictive analysis, the number of amino acids (AAs) of the BnXTH proteins ranged from 212 to 473, the relative molecular mass (MW) ranged from 24.42 to 55.10, and the theoretical isoelectric point (PI) ranged from 4.77 to 9.96 (Table S1). Subcellular localization prediction results showed that 54 XTH proteins may exist in the cytoplasm, 21 in the cell wall, and 5 in the nucleus.

### Phylogenetic analysis of BnXTH proteins

**Fig. 2:**
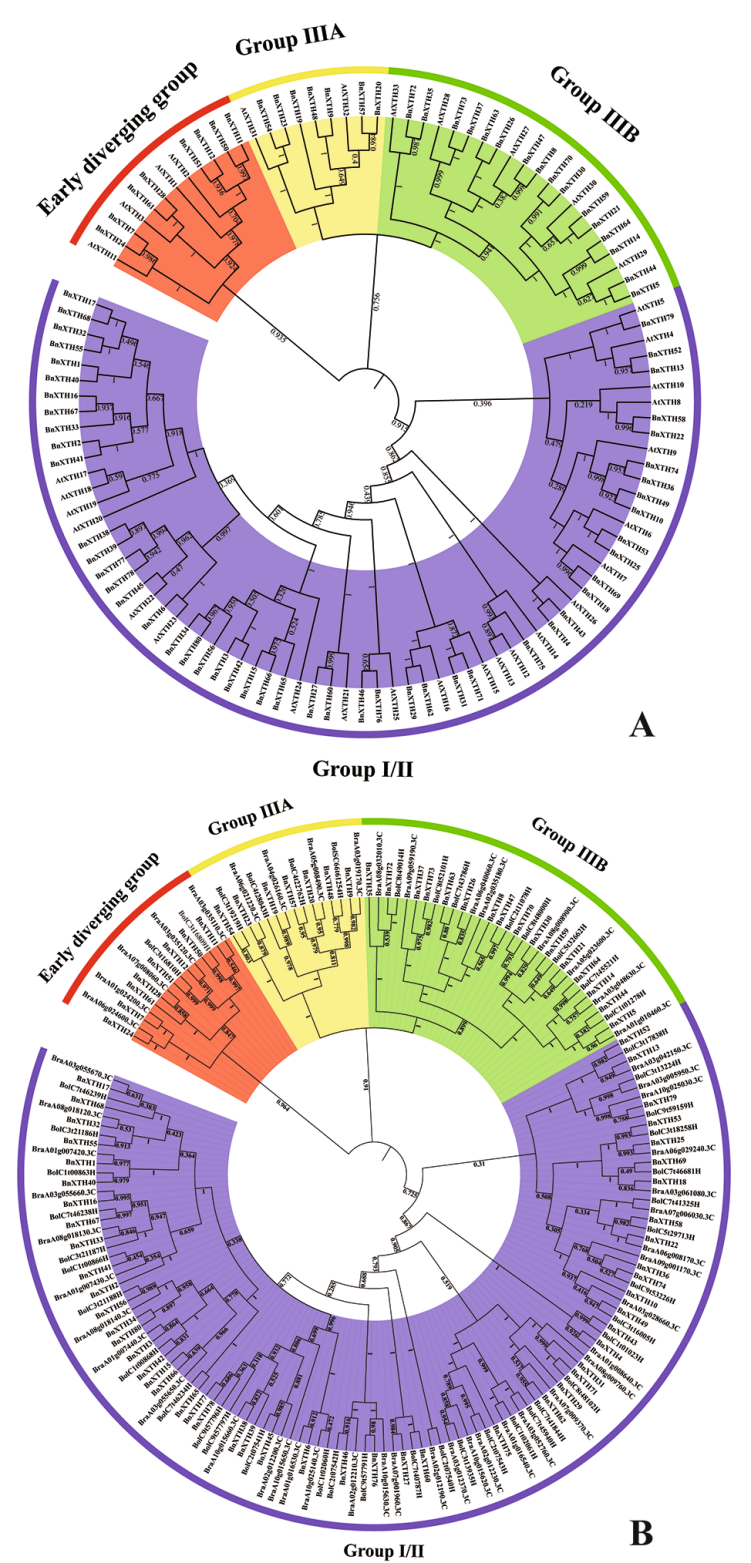
Phylogenetic tree of XTH proteins. (**A**) Phylogenetic tree of XTH proteins in *B. napus* and *(A) Thaliana*. (**B**) Phylogenetic tree of XTH proteins in *(B) napus*, *B. rapa*, and *B. oleracea*

In order to explore the phylogenetic relationship between XTH proteins, 80 XTH proteins of *B. napus* and 33 XTH proteins of *Arabidopsis thaliana* were combined to form a phylogenetic tree. Based on the genetic relationship between them and the position of the branch where the AtXTH proteins were located, XTH proteins were divided into four groups: Early diverging group, Group I/II, Group IIIA and Group IIIB (Fig. 2A). According to the phylogenetic tree, it could be seen that the number of proteins contained within each group varies, among which the most proteins were clustered into Group I/II, with 22 AtXTHs and 49 BnXTHs; in Group IIIB, there were 5 AtXTHs and 16 BnXTHs; in the Early diverging group, there were 4 AtXTHs and 8 BnXTHs; Group IIIA had the fewest proteins, with 2 AtXTHs and 7 BnXTHs (Table 1).

To further confirm the phylogenetic relationships of XTHs within Brassicaceae plants, a total of 52 XTH proteins from *Brassica rapa* and 47 XTH proteins from *Brassica oleracea* were identified by using BLASTP and HMMER methodologies for the construction of a phylogenetic tree (Table S2, Fig. 2B). The analysis revealed that these proteins could also be classified into four groups, with the groupings of BnXTHs remaining unchanged, thereby validating the reliability of the phylogenetic tree. Notably, the Group I/II contained the highest number of proteins, with 35 BrXTHs and 33 BoXTHs, followed by Group IIIB, which comprised 8 BrXTHs and 8 BoXTHs. Group IIIA included 4 BrXTHs and 4 BoXTHs, and the Early diverging group consisted of 4 BrXTHs and 4 BoXTHs (Table 1).

**Table 1:** Number of XTH proteins contained in each group in the phylogenetic tree

Group	Early diverging group	Group I/II	Group IIIA	Group IIIB
AtXTH Number	4	22	2	5
BnXTH Number	8	49	7	16
BrXTH Number	5	35	4	8
BoXTH Number	2	33	4	8

### Gene structures, conserved motifs and promoter cis-acting elements analysis of *BnXTHs*

**Fig. 3:**
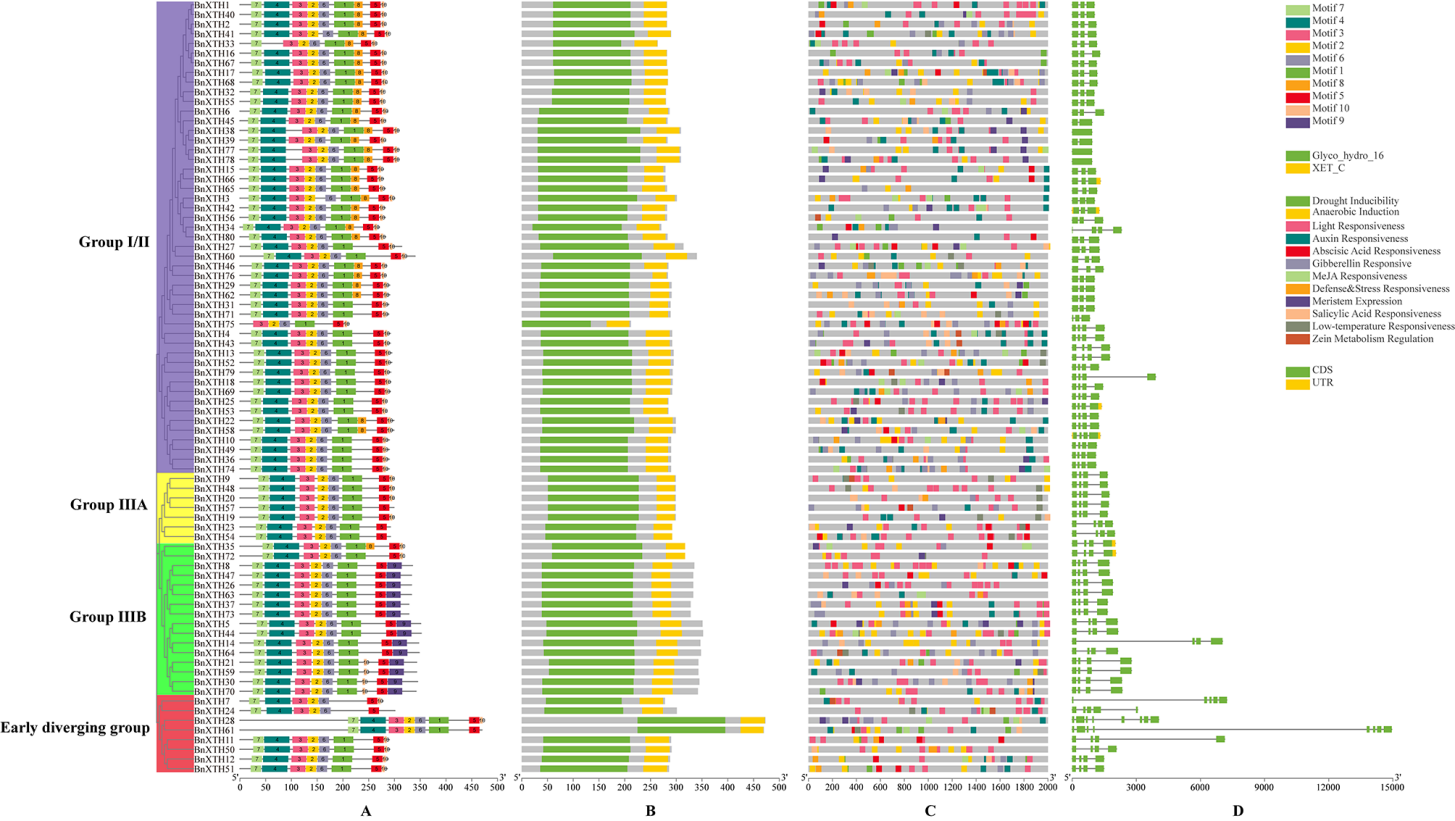
Gene structure analysis of *XTH* family in *B.napus*. (**A**) Conserved motifs of BnXTH family proteins. (**B**) Pfam structure of BnXTH family proteins. (**C**) Promoter cis-acting element of *BnXTHs*. (**D**) The mRNA structure encoded by the *BnXTHs*

Analysis of motifs in conserved domains is a powerful tool for understanding the function, structure, and evolution of proteins. Conserved motif analysis of BnXTH proteins shows that Motif2, Motif 3, Motif 5 and Motif 6 were common to all BnXTH proteins; Motif7 was merely absent from BnXTH75; Motif4 was absent from BnXTH33 and BnXTH75 of Group I/II; Motif1 was absent in BnXTH7 and BnXTH24 of the Early diverging group; most Group IIIB and a small number of Early diverging group proteins did not contain Motif10; Motif8 existed in some Group I/II and BnXTH35 proteins; Motif9 was present in most of the Group IIIB proteins. (Fig. 3A). The protein structural characteristics of BnXTHs were identified, and all proteins were found to contain a Glyco_hydro_16 and a XET_C domain (Fig. 3B). Analysis of promoter cis-acting elements showed that *BnXTHs* may have a response mechanism in response to stress and plant hormone signals (Fig. 3C). Among them, there were as many as 910 light responsiveness elements, far more than other cis-acting elements. In addition, *BnXTH41* and *BnXTH45* contained the most cis-acting elements, amounting to 41, indicating that they may play roles in the growth and development of rapeseed. The study of the mature mRNA structure of *BnXTHs* revealed that they contained 1–8 CDS (Coding sequence) regions (Fig. 3D), and some *BnXTHs* also contained 1–2 UTR (Untranslational region) regions.

### Collinearity analysis of *XTH* gene family

**Fig. 4:**
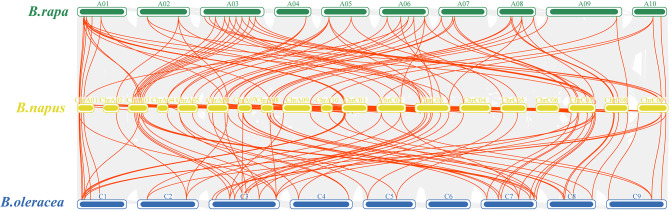
Collinearity of *XTH* genes in *B. napus*, *B. rapa*, and *B. oleracea*

**Fig. 5:**
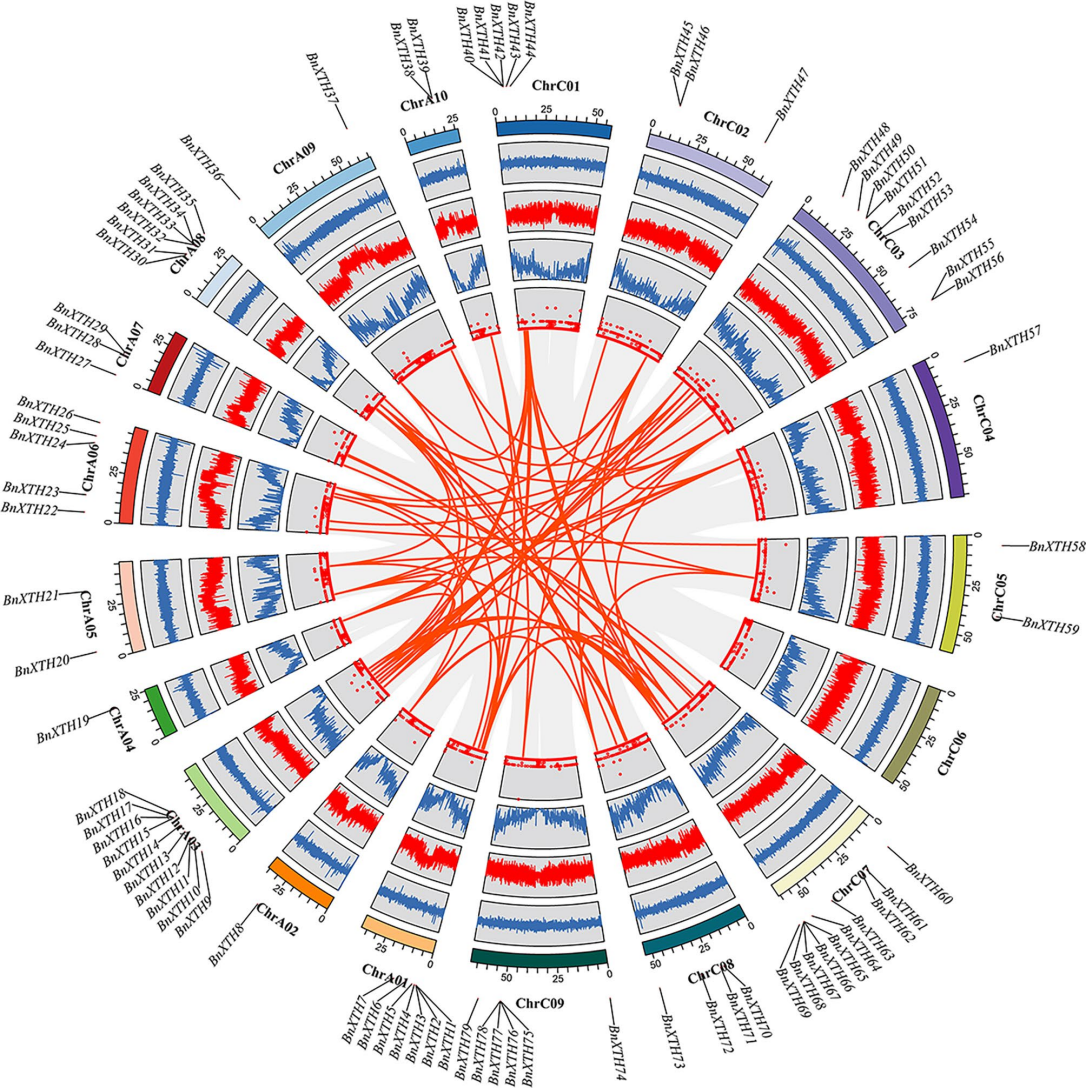
Collinearity of *BnXTH*s. The circles in the figure from inside to outside represent the unknown base N ratio, gene density, GC ratio, GC skew, and chromosome length of the *B. napus* genome

In order to investigate the collinearity of *XTH* genes, *BnXTH80* located on the pseudochromosome was removed and the collinearity maps of rapeseed (*B. napus*), *B. rapa* and *B. oleracea* were drawn (Fig. 4). There were 92 pairs of collinear genes between the *XTH* genes of *B. napus* and *B. rapa*, and 88 pairs of collinear genes between the *XTH* genes of *B. napus* and *B. oleracea* (Table S3). Collinearity analysis of *BnXTHs* within the *B. napus* genome showed that there were 83 pairs of collinear genes among *BnXTHs* (Fig. 5 and Table S4). To understand the genetic relationship of *BnXTH* collinear gene pairs, KaKs values were calculated to understand their selection pressure relationships (Table S5). The results showed that the Ka/Ks values of these 83 collinear gene pairs were all less than 1, indicating that they were all affected by Purification selection.

### Prediction of targeting relationship between miRNA and *BnXTHs*

**Fig. 6:**
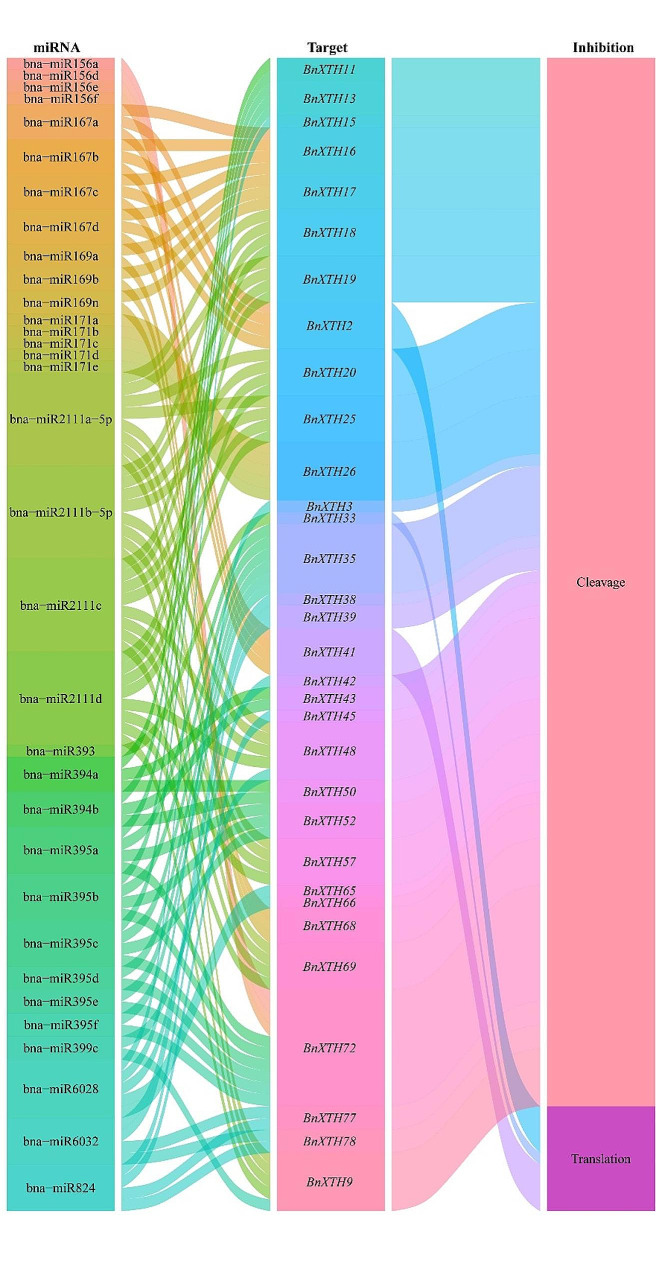
Sankey diagram for the relationships of miRNA targeting to *BnXTHs* transcripts. The 3 columns represent miRNA, *BnXTHs* and inhibition effect

As shown in Fig. 6, a total of 32 miRNAs of *B. napus* targeted 29 *BnXTH* genes through cleavage, and 5 miRNAs of *B. napus* target 3 *BnXTH* genes through translation. No miRNA was found to have both cleavage and translation inhibition effects on the same *BnXTH* gene. The results suggest that multiple miRNAs were involved in the post-transcriptional regulation of *BnXTHs* by targeting them through cleavage and translation.

### Analysis of transcriptome expression patterns of *BnXTHs* under abiotic stresses

**Fig. 7:**
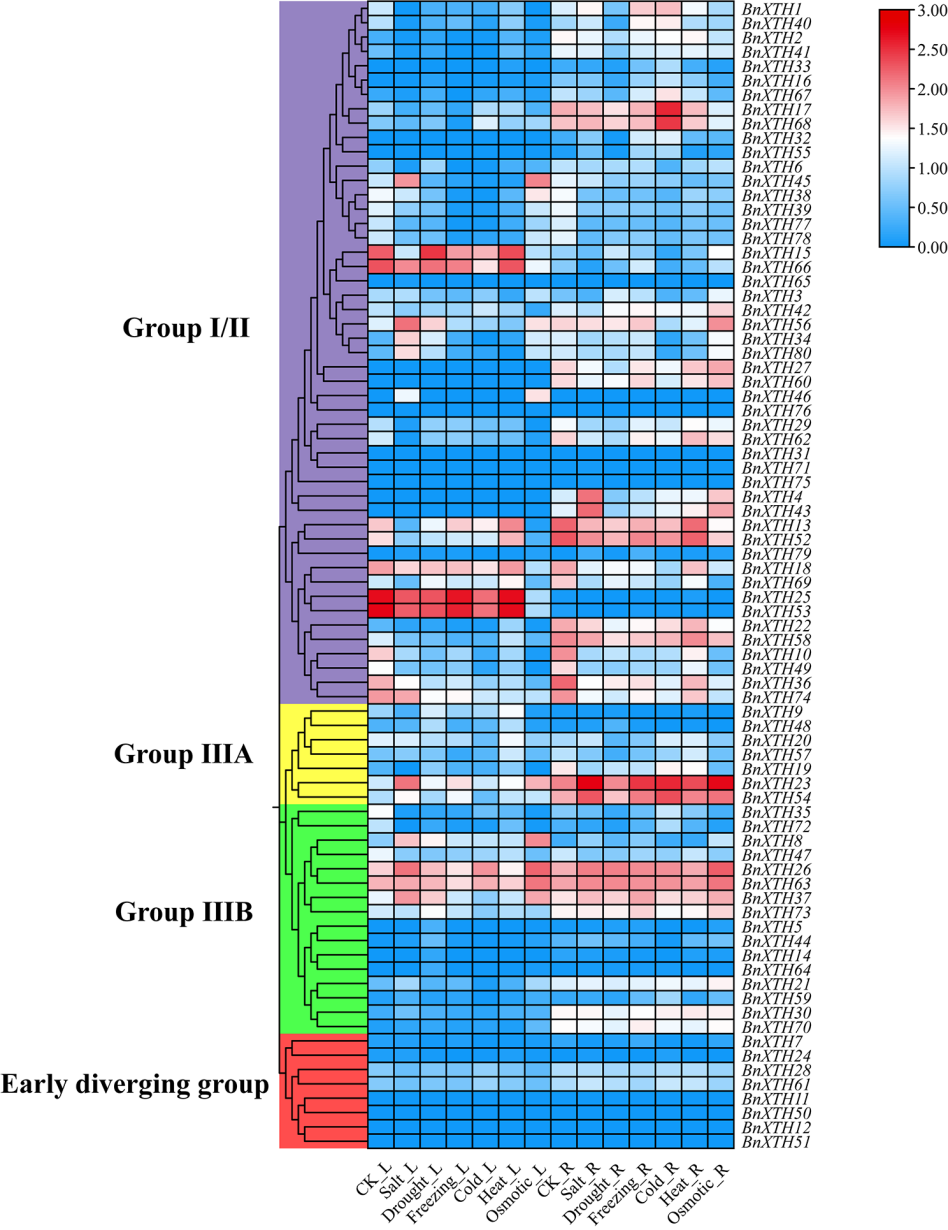
Analysis of expression patterns of *BnXTHs* under abiotic stress (CK, salt, drought, freezing, cold, heat, and osmotic) treatments. L: leaves; R: Roots

In order to explore the gene expression patterns of *BnXTHs* under different abiotic stresses, we obtained the transcriptome TPM data of *BnXTHs* in *B. napus* ZS11 under CK, salt, drought, freezing, cold, heat and osmotic stress, and expression heat maps were plotted after log_10_(TPM + 1) treatment (Fig. 7). Some *BnXTHs* were not expressed or had low expression in leaves and roots under different stresses; however, *BnXTH26*, *BnXTH63* and *BnXTH37* showed high expression in leaves and roots under various stresses; *BnXTH13* and *BnXTH52* had high expression in roots under various stresses and high expression in leaves under some of the stresses; the expression levels of *BnXTH15*, *BnXTH66*, *BnXTH25*, and *BnXTH53* were higher in leaves under various stresses; the expression levels of *BnXTH17*, *BnXTH68*, *BnXTH22*, *BnXTH58*, and *BnXTH73* showed higher expression in roots under various stresses.

### Detection of *BnXTHs* expression under various abiotic stresses by qPCR technique

**Fig. 8:**
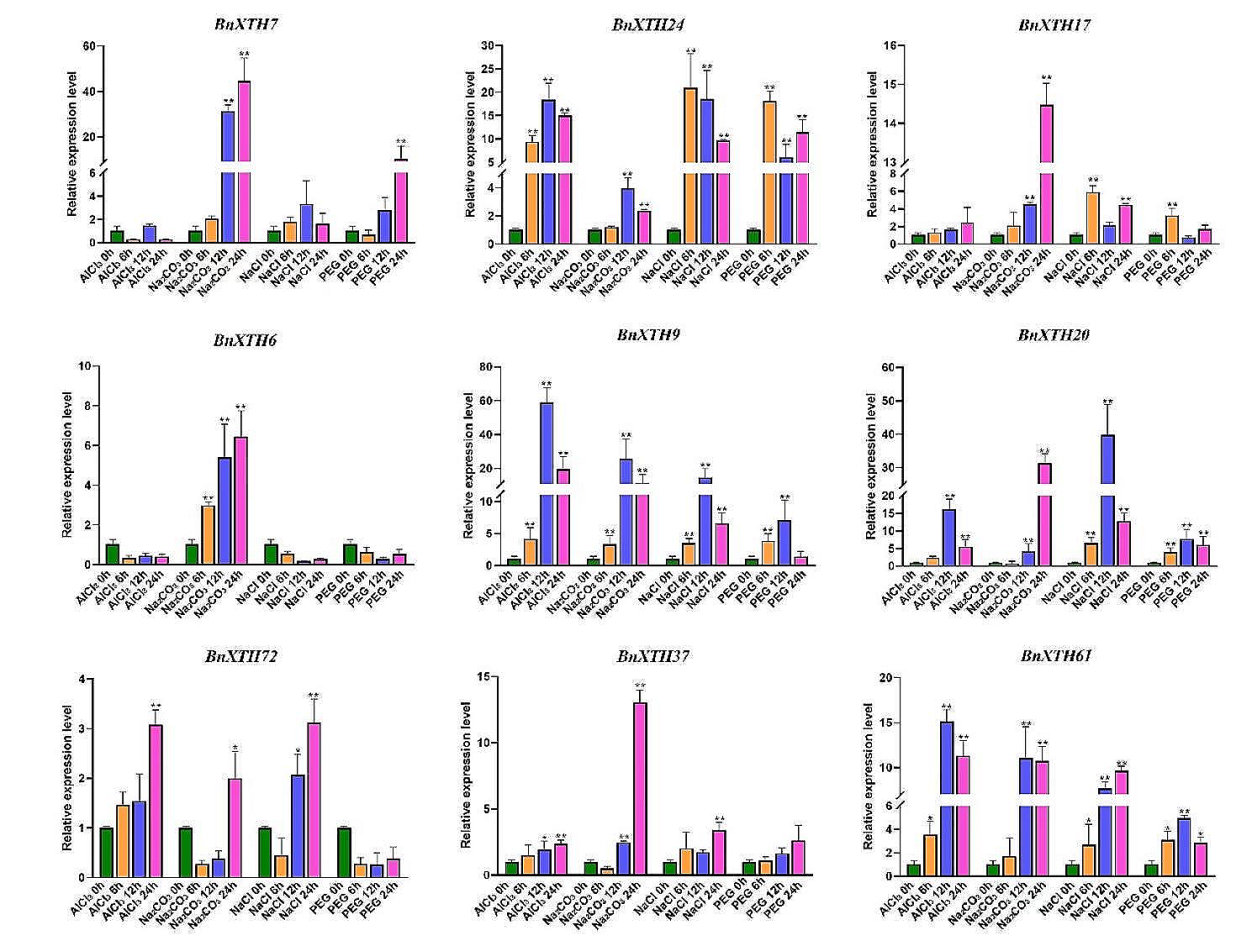
The relative expression of *BnXTH7*, *BnXTH24*, *BnXTH17*, *BnXTH6*, *BnXTH9*, *BnXTH20*, *BnXTH72*, *BnXTH37*, and *BnXTH61* in leaves under 0.5 mmol·L^-1^ AlCl_3_, 0.2% (w/v) Na_2_CO_3_, 1.2% (w/v) NaCl, and 20% (w/v) PEG 6000 after 0, 6 h, 12 h and 24 h. Data represent the mean ± standard error for threebiological experiments. Student’s t-test was used to determine differences. *: significant differences between treatments at *p* ≤ 0.05. **: significant differences between treatments at *p* ≤ 0.01

The cell wall is the first barrier to protect plant cells from damage, and the XTH genes plays an important role in plants defense against abiotic stress. Therefore, to investigate the effect of BnXTHs on rapeseed response to abiotic stress, we performed qPCR on leaves of oilseed rape seedling ZS11 treated with 0.5 mmol·L^-1^ AlCl_3_, 0.2% (w/v) Na_2_CO_3_, 1.2% (w/v) NaCl, and 20% (w/v) PEG 6000 after 0, 6 h, 12 h and 24 h to analyze the relative expression of 9 different BnXTHs (Fig. 8). The results showed that they responded significantly to different abiotic stresses.

Under AlCl_3_ treatment, the expression of *BnXTH7*, *BnXTH17*, and *BnXTH6* did not change significantly at all time points; *BnXTH72* and *BnXTH37* increased with the extension of the treatment time; *BnXTH24*, *BnXTH9*, *BnXTH20*, and *BnXTH61* increased with the extension of the treatment time, reaching the maximum at 12 h, and then decreased at 24 h again.

Under Na_2_CO_3_ treatment, the expression of *BnXTH7*, *BnXTH17*, *BnXTH6*, *BnXTH20*, and *BnXTH37* increased with the prolongation of the treatment time; the expression of *BnXTH24*, BnXTH19, and *BnXTH61* increased and then decreased; and the expression of *BnXTH72* showed a decreasing tendency first, but it was insignificant, and then increased significantly at 24 h. The expression of *BnXTH72* increased significantly at 24 h, but it was not significant.

Under NaCl treatment, the expression of *BnXTH7* and *BnXTH6* did not change significantly; the expression of *BnXTH72*, *BnXTH37*, and *BnXTH61* increased with the prolongation of time; and the expression of *BnXTH24*, *BnXTH17*, *BnXTH9*, and *BnXTH20* increased and then decreased.

Under PEG 6000-induced drought stress, the expression of *BnXTH6*, *BnXTH72*, and *BnXTH37* did not change significantly; the expression of *BnXTH7* increased with the prolongation of time; the expression of *BnXTH17*, *BnXTH9*, *BnXTH20*, and *BnXTH61* increased and then decreased; the expression of *BnXTH24* at 6 h and then decreased at 12 h. The expression of *BnXTH24* was elevated at 6 h, and then increased again at 24 h. The expression of *BnXTH24* increased at 6 h and then decreased at 12 h, and then increased again after 24 h.

## Discussion

Gene family analysis is conducive to mining key functional genes in crop genomes and provides a genetic research basis for the development of high-yield and high-quality germplasms. In this study, the Arabidopsis XTH protein sequences were used as the seed sequence, combined with the Pfam domain search results, and finally identified 80 members of the *B. napus XTH* gene family, distributed on 18 definite chromosomes and 1 pseudochromosome. In this study, 52 BrXTH proteins and 47 BoXTH proteins were identified, the numbers of which differ from previous studies (53 *XTHs* of *B. rapa* and 38 *XTHs* of *B. oleracea*) [21]. This discrepancy may be due to differences in the sources of genome databases and the selection of thresholds. Compared with the 52 BrXTH proteins and 47 BoXTH proteins, *B. napus*, as an allotetraploid, was identified with 80 *XTH* genes, a number smaller than the sum of the former two, which also suggested that recombination or mutation at the gene or chromosome level may have occurred in the *B. napus* genome during the evolutionary process. Phylogenetic analysis was performed based on the XTH protein sequences of *(A) thaliana* and *(B) napus*, and the distribution of XTH proteins in the phylogenetic tree was statistically analyzed. It was found that BnXTH proteins were more uniformly distributed among the four groups of the phylogenetic tree, suggesting that gene duplication events might have occurred in the genome of *B. napus* at the whole-genome level in these four groups (Fig. 2A). In the construction of phylogenetic trees for three species within the Brassicaceae family, it was observed that the distribution of XTHs within the four groups was broadly consistent (refer to Fig. 2B; Table 1). According to the U-triangle model, *B. napus* (AACC: 2n = 38), an allopolyploid species, originates from the cross between *B. rapa* (AA: 2n = 20) and *B. oleracea* (CC: 2n = 18) [22]. This analogous distribution pattern of XTHs across the species underscores their genetic conservation. Furthermore, the total count of BnXTHs being lesser than the combined total of BrXTHs and BoXTHs highlights the evolutionary distinctiveness of BnXTHs.

Further analysis of the gene structures and conserved domains showed that evolutionary conservation may exist among species including *(A) thaliana* [23], *Osmanthus fragrans* [24], *Schima superba* [25], *(B) rapa* and *B. oleracea* [21]. Motif 2, Motif 3, Motif 5, and Motif 6 were identified as ubiquitous across all BnXTH proteins. This uniform presence suggests that these four motifs likely constitute characteristic sequences of BnXTHs, playing pivotal roles in the structural and functional attributes of these proteins. Analysis of promoter cis-acting elements showed that the function of *BnXTHs* may be related to photosynthesis due to the large number of light responsiveness elements. By modifying the cell wall, XTHs influence leaf thickness, which can affect the internal leaf architecture and, consequently, the efficiency of light capture and the distribution of light within the leaf [16]. XTHs are also involved in the development and functioning of stomata by modulating the flexibility and integrity of the cell walls surrounding guard cells [26]. While the direct relationship between XTH activity and photosynthesis requires further empirical study, it is evident that XTHs, through their role in cell wall modification, indirectly contribute to optimizing the conditions necessary for efficient photosynthesis.

The Ka/Ks analysis is a method used to measure the selective pressure on genes, commonly applied in the study of gene or gene family evolution. In this context, Ka represents the rate of nonsynonymous substitutions, which lead to changes in amino acids, while Ks denotes the rate of synonymous substitutions that do not result in amino acid changes [27]. Ka/Ks analysis serves as a crucial tool for understanding the dynamics of gene evolution and revealing the key mechanisms behind the adaptive evolution of organisms, suitable for discussion in scientific literature. By collinearity analysis between genomes, we found that there were 92 pairs of *XTH* collinear genes between *B. napus* and *B. rapa*, 88 pairs of *XTH* collinear genes between *B. napus* and *B. oleracea* (Fig. 4), and 83 *XTH* covariance pairs within the *B. napus* genome (Fig. 5), and their Ka/Ks values were all less than 1 (Table S5). The number of collinear gene pairs of *XTHs* among species was greater than the number of *BnXTHs* we identified, indicating that *XTHs* are evolutionarily correlated and have both inter- and intra-species evolutionary conservation.

miRNAs (microRNA) are small non-coding RNAs that regulate gene expression at the post-transcriptional level by inhibiting the translation of messenger RNAs (mRNAs) or by promoting mRNA degradation [28]. miRNAs are approximately 20–24 nucleotides (nt) in size and are encoded by plants, animals and some viruses [29]. In plants, miRNAs inhibit gene expression mainly by mediating target RNA cleavage or translational repression [30], and they control target genes at the post-transcriptional or translational level by controlling the level of protein synthesis, and have an impact on plant growth, development, and response to environmental stresses [31]. For example, bna-miR159, bna-miR6029, and bna-miR827 negatively regulate target genes related to nitrogen metabolism pathway in *B. napus*, thereby affecting nitrogen signaling within the *B. napus* plant and consequently its pod thickness [32]. bna- miR319 overexpressing transgenic lines of *B. napus* exhibited abnormal development of serrated leaves and stem tip meristematic tissues [33]. Fu et al. analyzed miRNA-mRNA expression of Cd-stressed *B. napus* seedlings and found that Cd treatment significantly affected the expression of 22 miRNAs belonging to 11 families in roots and 29 miRNAs belonging to 14 families in shoots, and identified 8 miRNA-mRNA interaction pairs in roots and 8 miRNA-mRNA interaction pairs in shoots. 8 miRNA-mRNA interaction pairs in roots and 8 miRNA-mRNA interaction pairs in shoots were identified [34]. In this study, a total of 37 miRNAs of *B. napus* were targeted to 29 *BnXTH* genes through cleavage and translational repression (Fig. 6), and it was hypothesized that miRNAs form a miRNA-target gene regulatory network with *BnXTHs*, which are involved in the process of reorganization and hydrolysis of XG in *B. napus* cell walls. Interestingly, *BnXTHs* have similar cis-acting elements (Fig. 3), but could have relatively different expression patterns (Fig. 7). For example, *BnXTH2*, and *BnXTH41* all targetted bna-miR167 and had relatively consistent expression patterns. They were not expressed in leaves under various abiotic stresses but have a certain amount of expression in roots. However, compared with the expression patterns of other *BnXTHs*, they were different.

Various abiotic stresses may affect the integrity of plant cell walls, but plants are able to repair adverse changes in the cell wall, including changing its composition, structure, and mechanical properties to maintain growth [35]. There has been some research on how the cell wall responds to stress [36]. However, due to the constraints of imaging and biomechanical equipment and the potential crosstalk between cell wall signaling and stress signaling, it is difficult to draw general conclusions about the regulatory mechanisms of the cell wall in response to various abiotic stresses [2]. *XTH* genes are key genes that regulate the hydrolysis and recombination of XG components and plays an important role in the structure and composition of plant cell walls [25]. Therefore, clarifying the changes that occur in *XTHs* during plant defense against abiotic stresses is informative for the study of the plant stress regulatory mechanism mediated by plant cell wall signals. In this study, qPCR technology was used to detect the relative expression *BnXTH7*, *BnXTH24*, *BnXTH17*, *BnXTH6*, *BnXTH9*, *BnXTH20*, *BnXTH72*, *BnXTH37*, and *BnXTH61* in leaves under 4 abiotic stresses treatment after 4 time points: 0, 6 h, 12 h, and 24 h (Fig. 8). It was found that in response to abiotic stresses, the expression of most of the *BnXTHs* gradually increased or first increased and then decreased with the growth of treatment time, indicating that oilseed rape needs large amounts of recombinant XGs in response to abiotic stress in order to repair the damage that may be caused by the stress to the cell wall. And with the time went, the plant slowly adapted to the stress, and some of the *BnXTHs* may have received the relevant signals, thus the expression fell back.

## Materials and methods

### Identification and chromosome mapping of *BnXTH* family members

The sequence files of ZS11 (a variety of *B.napus*), cabbage (*B. rapa*), and kale (*B. oleracea*) were obtained from the BRAD database (BRAD: http://brassicadb.cn/#/) [37]. 33 AtXTH protein sequences from the *A. thaliana* genome database (TAIR: https://www.arabidopsis.org/) were downloaded as seed sequences, and searched for possible BnXTH proteins in the whole protein sequences of *B.napus* (e Value < 1e-10) by BLASTp [38]. The PF06955 and PF00722 conserved structural domain files were downloaded from the Pfam database to search the possible BnXTH proteins by HMMER software (http://www.hmmer.org/) [39, 40], combining the BLAST results to obtain the BnXTH hypothetical proteins. The putative protein sequence was uploaded to the NCBI-CDD website (https://www.ncbi.nlm.nih.gov/cdd/) for further confirmation, and 80 members of the *BnXTH* gene family were finally identified. The *BnXTHs* were named according to their chromosomal position sequence, and were named as *BnXTH1* ~ *BnXTH80*. The Protparam function in the ExPASy website was used to predict the physical and chemical properties of BnXTH proteins [41], and the subcellular localization prediction results were implemented by the Plant-mPLoc website [42]. Chromosomal location information of *BnXTHs* was retrieved using downloaded annotation files and visualized by TBTools [43].

### Phylogenetic analysis

The XTH protein sequences of *A. thaliana* and *B.napus* were imported into MEGA 11 software, and the parameters were adjusted to neighbor joining (NJ) and 1000 boots-trap repetitions to obtain a phylogenetic tree, and the phylogenetic tree was embellished in the online website iTOL (https://itol.embl.de/) [44, 45].

### Prediction of gene structure, protein conserved motifs and promoter cis-acting elements

The CDS/UTR region and gene structure information of *BnXTH* gene family members were obtained from the NCBI-CDD website and Pfam database. The MEME 5.5.1 website was used to obtain the conserved motif of the BnXTH proteins [46], and the prediction of the cis-acting element 2000 bp upstream of the BnXTHs promoter region was predicted through the PlantCARE website [47]. All data information visualization was completed by TBTools.

### Collinearity analysis of *XTH* genes

MCScanX software was used to analyze the collinear relationship between *XTHs* within the genomes of *B. napus, B. rapa*, and *B. oleracea*, as well as within the genomes of *B. napus* [48]. Collinearity information visualization was performed using TBTools. Ka/Ks value calculation was implemented using KaKs_calculator 3.0 software [49].

### Prediction of targeting relationship between miRNA and *BnXTHs*

The CDS sequences of *BnXTHs* were uploaded to the psRNATarget website (https://www.zhaolab.org/psRNATarget/analysis?function=2/) [50], and combined with the miRNA mature sequences information of *B. napus* collected by the website, the targeting relationships between miRNA and *BnXTHs* were analyzed. The data were visualized using the Bioinformatics website (https://www.bioinformatics.com.cn/plot_basic_alluvial_plot_017).

### Analysis of the expression pattern of *BnXTHs* under different abiotic stress

To investigate the gene expression patterns of *BnXTHs* under abiotic stress treatments, the *BnXTH* gene IDs were uploaded to the BNIR database [51] to obtain the Transcript per Kilobase per Million mapped reads (TPM) of *B. napus* ZS11 in control (CK), salt (200 mmol·L^-1^ NaCl treatment), drought (exposure to airflow for 1 h), freezing (recovering to 25 °C after 3 h of stress at -4 °C), cold (4 °C), heat (recovering to 25 °C after 3 h of stress at 38 °C), and osmosis (300 mmol-L-1 mannitol) after 24 h, and the data were visualized using TBTools software after calculating log_10_(TPM + 1).

### Materials and treatment

*B. napus* ZS11 seeds were germinated on wet gauze (soaked with water) in a plant growth chamber at 20 to 22 °C and 65% humidity under a long-day condition (16-h-light/ 8-h-dark cycle) [52]. The one-week-old seedlings were then transferred into a previously described hydroponic system [53] under the same culture conditions for nearly 20 days until the fourth leaves had extended (plant samples used for transcriptome sequencing were also harvested for root system). For stress treatment research, leaf samples from 4-week-old plants of ZS11 were collected after 0, 6, 12 and 24 h of 0.5 mmol·L^-1^ AlCl_3_, 0.2% (w/v) Na_2_CO_3_, 1.2% (w/v) NaCl, and 20% (w/v) PEG 6000 treatment. Seedlings without any stress treatment were used as the control. Each treatment includes three biological replications. Leaves were harvested immediately frozen in liquid nitrogen and stored at -80 °C for RNA extraction.

### Relative expression of *BnXTHs* under different abiotic stresses

Total RNA of ZS11 under control and abiotic treatments was extracted using the RNA simple Total RNA kit (Tiangen Biotechnology Co., Ltd., Beijing, China) according to the manufacturer’s protocol. cDNA was synthesized with 1 µg RNA from each sample with HiScript® II Q Select RT SuperMix with gDNA wiper (Vazyme, Nanjing, China). Gene-specific primers used for quantitative real-time PCR (qRT-PCR) listed in Table 2. qRT-PCR was run on the AriaMx real-time PCR system (Agilent Technologies). The following cycling parameters were used: initial denaturation at 95 °C for 5 min; 40 amplification cycles consisting of denaturation at 95 °C for 10s, annealing and extension at 60 °C for 30 s; The melting curve was then tested at 65–95 °C. The internal standard was the *B. napus* actin gene (*BnaA01g27090D*). To investigate the expression patterns of *BnXTHs* under various environmental conditions, 9 *BnXTHs* were randomly selected from the 4 groups for qRT-PCR experiments. Three biotic replicates were performed for each sample, and each replicate contained three technical replicates. Relative expression levels were calculated according to the 2^−ΔΔCt^ method [54]. Data represent the mean ± standard error for threebiological experiments. Student’s t-test was used to determine differences. *: significant differences between treatments at *p* ≤ 0.05. **: significant differences between treatments at *p* ≤ 0.01.

**Table 2:** qPCR primer sequence of *BnXTH* genes

Gene Name	Forward Primer (5’→3’)	Reverse Primer (3’→5’)	Amplicon Length (bp)
*BnXTH6*	AAACGGGAAGTCATCGTGCT	CTGCACCCATCTCATCCGTT	109
*BnXTH7*	GGTGCAGACGATGTTCATGC	AAACTCAGTCGCACACCCAT	118
*BnXTH9*	GGGAAGTGGTGATGGCAGAA	GAAATGTGGCCGCACTCTTC	178
*BnXTH17*	ACTGCATCACATGGAACCCC	GCTCAGCTTCCCAAAGGCTA	114
*BnXTH20*	CTGGCTACACTGCTGGAGTC	GCGAAACTTCATCTCGCGAC	188
*BnXTH24*	ACCGATCCGAGTGTACAGGA	GATGCCTGGAACTCAGCAGT	135
*BnXTH37*	AGGTTCTGAGCTGCCAAGAC	CTAAGCCGCTTAGCCTCCTC	190
*BnXTH61*	GGACTTACGACAGGGAGCAG	AACGTCCAGAGCACCTCAAG	115
*BnXTH72*	TCGCCAAACTCACTCTCGAC	AACCACAACACCAGAGGCAA	122

## Conclusions

*B. napus* genome was identified to contain 80 *XTH* genes distributed on 18 definite chromosomes and one pseudochromosome. Their phylogenetic relationships, protein physicochemical properties, subcellular localization, gene structures, promoter cis-acting elements, covariance relationships, and reciprocal miRNAs were predicted and analyzed, and their transcriptional expression patterns as well as differences in expression in response to abiotic stress treatments were investigated. The results showed that the expression patterns of *BnXTHs* under abiotic stress treatments were varied, suggesting that cell wall signaling in *B. napus* in response to various abiotic stresses changes depending on the type of stress. The analysis of the *BnXTH* gene family and the study of the response pattern in abiotic stresses provide a good theoretical basis for further research on this family of genes in resistance breeding of *B. napus*.

### Electronic supplementary material

Below is the link to the electronic supplementary material.

Supplementary Material 1: Table S1: Physicochemical properties and subcellular localization prediction of BnXTH proteins.

Supplementary Material 2: Table S2: Table S2. XTH IDs of *B. rapa* and *B. oleracea*.

Supplementary Material 3: Table S3: Collinearity of *XTH* genes in *B. napus*, *B. rapa*, and *B. oleracea*.

Supplementary Material 4: Table S5: Collinearity of *XTH* genes in *B. napus*.

Supplementary Material 5: Table S4: Collinearity of *XTH* genes in *B. napus*.

## Data Availability

All the data generated or analyzed during this study are included in this published article and its supplementary information files.
